# FLAIR Hyperintense Cortical Lesions in a 4-Year-Old Child with Myelin Oligodendrocyte Glycoprotein (MOG)-Associated Encephalitis and Seizures: A Case Report

**DOI:** 10.3390/children11070778

**Published:** 2024-06-27

**Authors:** Luca Bernardi, Nicole Mussi, Roberto Grandinetti, Emanuela Turco, Benedetta Piccolo, Francesca Ormitti, Nicola Principi, Susanna Esposito

**Affiliations:** 1 Pediatric Clinic, Department of Medicine and Surgery, Azienda Ospedaliero-Universitaria di Parma, 43126 Parma, Italy; bernardi.luca91@gmail.com (L.B.); nicole.mussi@unipr.it (N.M.); robertograndinetti93@gmail.com (R.G.); eturco@ao.pr.it (E.T.); bpiccolo@ao.pr.it (B.P.); 2 Unit of Neuroradiology, Azienda Ospedaliero-Universitaria di Parma, 43126 Parma, Italy; formitti@ao.pr.it; 3 Università degli Studi di Milano, 20122 Milan, Italy; nicola.principi@unimi.it

**Keywords:** encephalitis, FLAMES, MOG antibodies, pediatric neurology, seizures

## Abstract

Myelin oligodendrocyte glycoprotein (MOG)-IgG-associated disease (MOGAD) is a relatively uncommon autoantibody demyelinating disorder of the central nervous system (CNS) with heterogeneous clinical manifestations and magnetic resonance imaging (MRI) findings. In recent years, a rare MOGAD subtype characterized by distinct clinical and MRI findings has been described. Seizures and cortical hyperintensities best seen on MRI T2-weighted fluid-attenuated inversion recovery (FLAIR) sequences, associated with headache and cerebral spine fluid (CSF) pleocytosis, are the most important characteristics of this MOGAD entity that is named FLAMES (FLAIR hyperintense cortical lesions in MOG-associated encephalitis with seizures). Because of its rarity and the peculiarities of the brain damage and clinical manifestations, it can be under-recognized and confused with focal viral encephalitis, meningitis, subarachnoid hemorrhage, CNS vasculitis, or mitochondrial cytopathy. We described the case of a 4-year-old previously healthy girl who was admitted for focal-onset, tonic-clonic seizures, fever, and headache, combined with optic neuritis. MRI was characterized by FLAIR imaging showing hyperintense cortical lesions, and a mild leukocytosis in the CSF was detected. Efficacy and rapid response to steroid therapy was observed, and no recurrences of neurological problems or further seizures were reported in the following 12 months. This case report can help in understanding FLAMES characteristics in pediatrics in order to favor early diagnosis and prompt therapy.

## 1. Introduction

Myelin oligodendrocyte glycoprotein (MOG)-IgG-associated disease (MOGAD) is a relatively uncommon autoantibody demyelinating disorder of the central nervous system (CNS) [1]. Compared to other CNS demyelinating diseases, MOGAD has significantly more heterogeneous clinical manifestations and magnetic resonance imaging (MRI) findings that can occur in isolation or in various combinations. Optic neuritis, spinal cord damage, and ADEM-like disease are the most common clinical manifestations of MOGAD; however, brainstem and peripheral nervous system involvement have been reported [2]. In recent years, an even rarer MOGAD subtype characterized by distinct clinical and MRI findings has been described [3,4]. Seizures and cortical hyperintensities best seen on MRI T2-weighted fluid-attenuated inversion recovery (FLAIR) sequences associated with headache and cerebral spine fluid (CSF) pleocytosis are the most important characteristics of this MOGAD entity. This MOGAD subtype is named FLAMES (FLAIR hyperintense cortical lesions in MOG-associated encephalitis with seizures). Because of its rarity and the peculiarities of the brain damage and clinical manifestations, it can be under-recognized and confused with focal viral encephalitis (herpes); meningitis; subarachnoid hemorrhage; CNS vasculitis; and mitochondrial cytopathy, especially MELAS (mitochondrial encephalopathy, lactic acidosis, and stroke-like episodes) [3,4]. This can significantly delay diagnosis and prescription of an effective treatment, potentially causing evolution into status epilepticus, requiring ventilatory support, recurrences, and persistent brain alterations, as reported for other types of MOGAD [5]. Although 40 to 50% of MOGAD patients suffer from a single episode and totally heal after prompt therapy, the remaining patients experience one or more recurrences, and some of them present persistent brain alterations that suggest the importance of relapse prevention [6]. 

Unfortunately, reported cases of FLAMES are too few to allow a precise definition of this disease. Clinical characteristics and long-term prognosis of this MOGAD subtype are not precisely defined, and it is not known whether age and sex play a role in favoring the disease development and outcome. This case report can help in understanding FLAMES characteristics and favor early diagnosis and prompt therapy. 

## 2. Case Presentation

A 4-year-old previously healthy girl was admitted to the Emergency Room (ER) of the hospital due to a previous seizure episode of unknown duration, characterized by consciousness loss, deviation of the gaze and the head to the left, trismus, and hypertonia of the limbs. The episode was preceded by 10 days of frontal headache, abdominal pain, and mild fever (maximal axillary temperature, 37.5 °C) and resolved after administration of intravenous midazolam. At admission, the body temperature was in the normal range and the neurological examination was normal. No lumbar puncture was performed. Complete blood cell counts revealed neutrophilic leukocytosis (white blood cell count [WBC] 37,170/µL, neutrophils [N] 30,630/µL) and mild alteration of nonspecific inflammation markers (C-reactive protein [CRP] 13 mg/L, procalcitonin within the normal range).

**Figure 1:**
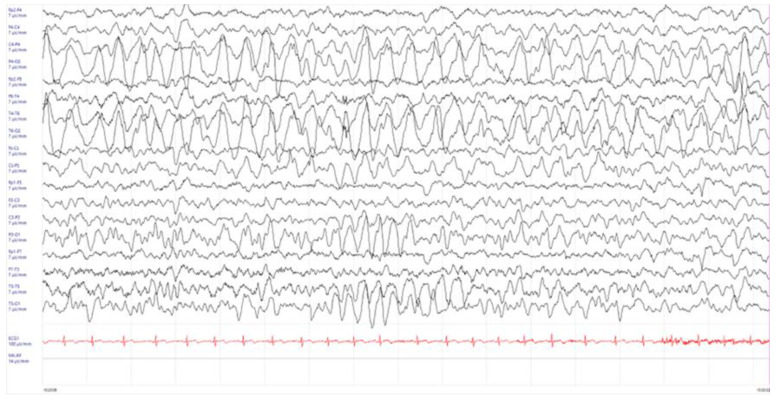
Electroencephalogram (EEG) performed one day after the occurrence of the generalized seizure. High amplitude slow delta activity in wake, over parietal-occipital regions, not reactive to eye opening was observed.

The electroencephalogram (EEG) showed disseminated slowing predominantly in the medium-posterior regions of the right hemisphere (Figure 1). 

**Figure 2:**
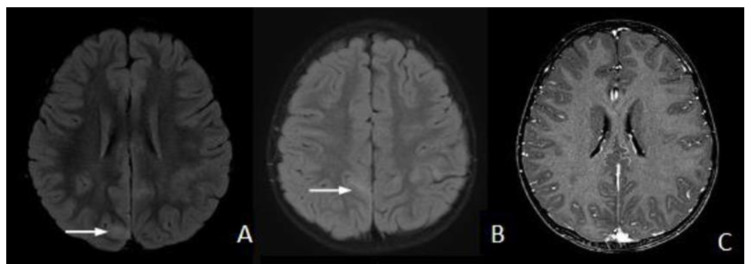
Magnetic resonance imaging (MRI) of the brain on admission (fluid-attenuated inversion recovery image is shown (**A**,**B**)). Slight swelling was seen mostly in the right parietal lobe, with a high-signal intensity area along cortex (arrow) and adjacent subcortical white matter. Axial T1-weighted post-gadolinium imaging (**C**) did not show contrast enhancement.

Brain MRI revealed narrowing of the sulci and cortical hyperintensities mostly throughout the cortex of the right parietal lobe on FLAIR (Figure 2A,B). No gadolinium-contrast enhancement was seen (Figure 2C). 

MRI alterations were initially ascribed to prolonged seizures status (PSS) or infectious/inflammatory disease/encephalitis. However, because neurological examination remained totally normal, and the neutrophilic leukocytosis was getting smaller (WBC 14,840/µL, N 10,900/µL), the girl was discharged from the hospital, and the follow-up was continued in a day-hospital setting.

**Figure 3:**
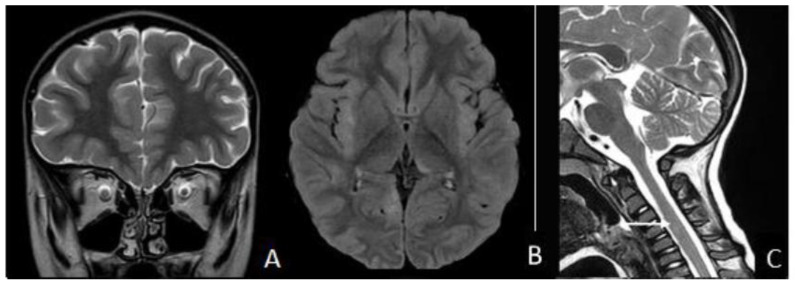
In a follow-up brain MRI acquired 1 week after first presentation, there is progression with right optic nerve T2 hyperintensity (**A**); bilateral multifocal lesions involving the cortical and subcortical areas, with mild mass effect on the adjacent sulci (fluid-attenuated inversion recovery [FLAIR] sequence; focal, unilateral, hyperintense lesions involving the left thalamus (**B**) and hyperintense lesion of the cervical cord (arrow (**C**)).

Nine days later, the patient was admitted again to the ER, presenting visual impairment and gait disturbance with ataxia. Eye examination showed no abnormalities. Brain MRI revealed right optic nerve T2 hyperintensity (Figure 3A) and multifocal white matter lesions in the cortico-subcortical white matter, extended to the left thalamus (Figure 3B), without contrast enhancement, suggestive of acquired CNS demyelinating disorder. Spinal cord MRI showed a hyperintense signal in the cervical cord, further suggesting the same diagnosis (Figure 3C).

Therefore, a lumbar puncture was performed with leakage of limpid cerebrospinal fluid (CSF) at normal pressure. Biochemical analyses of the CSF showed a mild increase in cells (12 leukocytes/µL) and normal values of cells, protein, and glucose. No oligoclonal bands were detected, and Link’s index was negative. Microbiological examination of the CSF ruled out viruses and bacterial infections. The CSF autoimmune encephalitis panel, including anti-NMDAR, gamma-aminobutyric acid (GABA) receptor, α-amino-3-hydroxy-5-methyl-4-isoxazolepropionic acid (AMPA) receptor, anti-leucine-rich glioma inactivated 1 (LGI-1) antibody, anti-contactin-associated protein-like 2 (CASPR2) antibody, DPPX, anti-aquaporin-4 (AQP-4) antibody, and anti-MOG antibodies, was negative. Serum samples were found positive for anti-MOG antibodies and negative for anti-aquaporin-4 (AQP-4) antibodies. The EEG continued to show disseminated slowing predominantly in the medium-posterior regions of the right hemisphere. 

According to the clinical history, laboratory investigation, and MRI scan findings, FLAMES was diagnosed, and therapy with a high dose of intravenous methylprednisolone (30 mg/kg/day) for 5 days tapering with oral prednisone (1 mg/kg/die) for 28 more days was prescribed. No antiepileptic therapy was administered.

**Figure 4:**
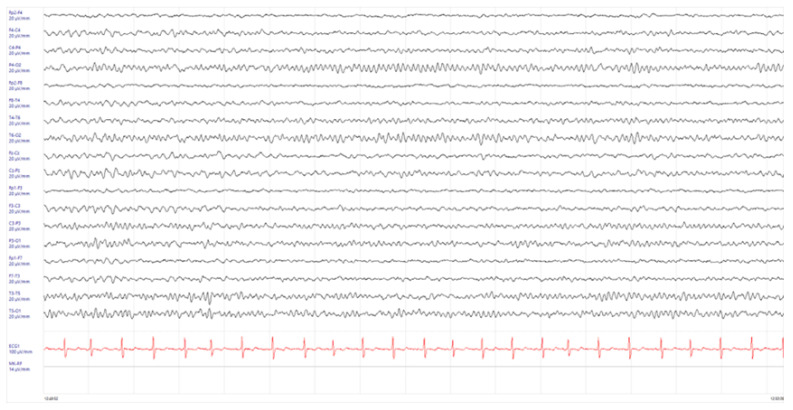
Electroencephalogram (EEG) performed 1 month after the start of immunosuppressive therapy in absence of antiepileptic treatment. No background activity alterations were described.

At the follow-up visit, 1 month after the start of immunosuppressive therapy, a neurological examination showed no abnormalities, and the EEG was found normal (Figure 4). 

**Figure 5:**
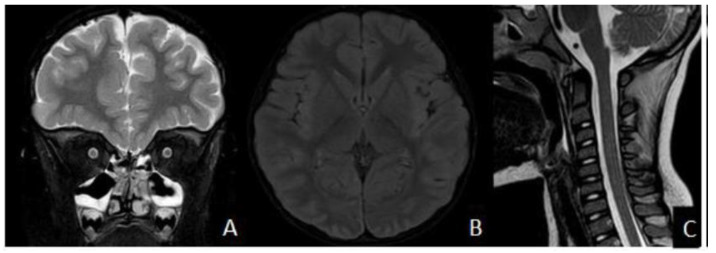
On axial T2-weighted fluid-attenuated inversion recovery (T2-FLAIR B) image pre-gadolinium, near-complete resolution of the previously demonstrated cortical and sulcal hyperintensity is seen (**A**), and there is a full recovery of the optic nerve (**B**) and cervical cord lesions (**C**).

Brain MRI with gadolinium 1 week later showed persistent cortical FLAIR hyperintensity within the frontal, parietal, and temporal lobes bilaterally (Figure 5). These areas appeared remarkably less conspicuous than previously noticed. No enhancement was seen after the administration of intravenous gadolinium. 

No recurrences of neurological problems or further seizures were reported in the following 12 months.

## 3. Discussion

FLAMES is a rare subtype of MOGAD clinical-radiological spectrum that differs from other MOG antibody-associated CNS diseases because of specific clinical, laboratory, and radiological characteristics. It was first described in 2017 by Ogawa et al. in four MOG antibody-positive adult patients presenting with generalized epileptic seizures with or without abnormal behavior or impaired consciousness [7]. In two cases, unilateral optic neuritis was also diagnosed. Laboratory tests were negative for encephalitis-associated autoantibodies but revealed moderate cerebrospinal fluid (CSF) mononuclear pleocytosis in all patients. Moreover, all patients had unilateral cortical/subcortical hyperintensities in white matter regions on brain magnetic resonance imaging (MRI) T2-fluid-attenuated inversion recovery (T2-FLAIR) sequences. FLAMES was considered a relatively benign condition, as administration of high-dose methylprednisolone was significantly effective. All patients fully recovered, abnormalities on the brain MRI disappeared, and no relapse occurred [7]. However, already from this report, it has been highlighted that diagnosis of FLAMES could be very difficult due to the similarities of signs and symptoms of this condition with other CNS diseases. Epileptic seizures and CSF pleocytosis are characteristic of viral meningoencephalitis. FLAIR-hyperintense lesions in the cerebral cortex or sulcus can occur in several CNS diseases including meningitis, subarachnoid hemorrhage, and acute infarction [8]. Diagnostic uncertainty, especially in the presence of fever, could occur. For this reason, treatment can be delayed or never prescribed, and a negative evolution is potentially possible. Further details on FLAMES clinical and radiological characteristics capable of favorable disease identification were reported by Budhram et al. in 2019 [9]. These authors described a total of 20 cases, including only one child, highlighting that most patients with this MOGAD subtype had fever, headache, seizures, hemiparesis, and lethargy. Moreover, on MRI, in most cases, unilateral cortical FLAIR hyperintensity in the right temporal lobe could be frequently evidenced without a sulcal FLAIR hyperintensity or meningeal enhancement. However, in 20% of the cases, MRI abnormalities were bilateral. Serum anti-MOG antibodies were systematically found. Treatment with steroids or immunosuppressive agents was always effective [9]. A more recent review by Wang et al. was able to quantify a prevalence of the different clinical manifestations of FLAMES [4]. It reported that seizures, headache, fever, and other cortical symptoms could be documented in 100%, 71.4%, 52.3%, and 61.9% of the cases, respectively. The common seizure types in FLAMES were focal to bilateral tonic-clonic seizures [10,11]. The cortical abnormalities on MRI FLAIR imaging were located mainly in the frontal, parietal, and temporal lobes. Generally, even in patients receiving immunosuppressive therapy, cortical abnormalities on MRI persist some months after the disappearance of clinical manifestations [12]. Pleocytosis in the CSF was documented in 95.2% of patients. The presence of anti-MOG antibodies was a constant; however, it is not definitively established whether titer of antibodies at disease onset was associated with severity of clinical manifestations. Finally, it was evidenced that about one third of patients were at risk of recurrence despite initially effective therapy, suggesting the need for long-term monitoring of all patients [10,11]. In this regard, accurate evaluation of anti-MOG antibody serum levels seemed to play a relevant role, as some data seemed to indicate that persistent detection of antibodies was a marker of risk or recurrence [13]. Despite these details, diagnosis of FLAMES remains complicated because this MOGAD subtype can coexist with anti-N-methyl-D-aspartate receptor encephalitis (anti-NMDARe), as repeatedly described [14,15,16]. This association can cause the development of an overlap syndrome with unknown clinical features and prognosis. However, according to the findings of the study by Yang et al., it can be suggested that cases with bilateral cortical FLAIR hyperintensity involving the medial frontal lobes are those with combined disease etiologies and those at increased risk of recurrences [15]. 

Studies have shown that immunosuppressive therapy is highly effective in FLAMES patients. Treatment is mandatory even in milder cases; however, some case reports have shown that FLAMES can spontaneously resolve [17]. This is because untreated patients may present persistent seizures or headache prior to resolution [18]. Suggested first-line treatment consists of high-dose corticosteroids (methylprednisolone, usually at 20–30 mg/kg/day to a maximum of 1 g/day) for 3–5 days before moving to corticosteroids orally administered (prednisone, 1–2 mg/kg/day) with tapering in no less than 5–12 weeks (shorter cycles are associated with a higher risk of relapses) [16]. Second-line treatment, instead, involves intravenous immunoglobulins (IVIg, maximum 2 mg/kg to be divided into 2–5 days) and plasmapheresis, which can be considered in severe cases and those nonresponding to the previous treatment lines. [19,20]. Immunosuppressive therapy can be considered as a subsequent step in the treatment: first of all, azathioprine is highly effective but slow in reaching an adequate level in therapeutic ranges; secondly, mycophenolate mofetil (MMF) has a quicker effect but should be reserved for cases with a poor response to azathioprine (at the present time, no reports about other immunosuppressors have a clinical impact) [21]. In our case, no second-line treatment was needed.

Most of the clinical, laboratory, and MRI findings characteristic of FLAMES diagnosis have been collected in adults; data regarding clinical manifestations of FLAMES in children are few. However, some reports seem to indicate that these are quite similar regardless of age and are mainly characterized by focal-onset, tonic-clonic seizures, fever and headache, combined with optic neuritis, as in the case described here. The risk of recurrences in children seems lower than in adults; however, the short follow-up of reported cases does not allow for firm conclusions to be drawn in this regard [22]. Moreover, in adults and children, MRI is characterized by FLAIR imaging showing hyperintense cortical lesions and a mild leukocytosis in the CSF. These findings suggest that the etiopathogenesis of FLAMES manifestations are common between pediatric and adult cases; however, further studies are needed for a complete understanding of the underlying phenomena. However, this case is fully representative of how difficult the diagnosis of FLAMES can be, especially when MRI findings in a child with previous seizures can be ascribed to previous protracted seizures or viral encephalitis. Moreover, this case shows that FLAMES, when properly treated, can resolve without CNS damage. Steroid therapy was promptly effective. The use of a second-line treatment was not needed. Clinical monitoring of the patient revealed a total resolution of the clinical manifestation of FLAMES signs and symptoms within one month from the end of therapy. MRI findings were significantly reduced, suggesting the arrest of the demyelination process and large brain renovation activity. 

## 4. Conclusions

This case report can help in understanding FLAMES characteristics in pediatrics in order to achieve early diagnosis and prompt therapy. The coexistence between cortical encephalitis with seizures and MOG antibodies is an emerging and challenging association; understanding its pathogenesis (from one side, neuronal cell dysfunction and from the other side, MOG antibodies, which selectively target oligodendrocytes) requires further studies and long-term follow-up, especially if it occurs in childhood.

## Data Availability

All the data are included in the manuscript.
